# When presence remains

**DOI:** 10.1017/S147895152610251X

**Published:** 2026-04-28

**Authors:** Miguel Augusto-Rodrigues

**Affiliations:** Faculty of Medicine of Ribeirão Preto, University of São Paulo, São Paulo, Brazil

The room was quiet, but not empty. There was a density to it – something that extended beyond illness. Not only the weight of disease, but of something medicine often struggles to name: the end.

He was 83 years old.

His chart was extensive, layered with diagnoses that had gradually transformed his body into a terrain of successive failures: heart disease, chronic lung disease, a malignancy no longer considered curable. From a clinical standpoint, he was a convergence of organ dysfunctions. But in that moment, he was simply a man breathing with effort – drawing each breath with a persistence that felt almost deliberate.

I sat beside him. I was part of a student initiative designed to visit older patients, to bring conversation into a space often defined by silence. But what unfolded there did not belong to any structured purpose.

His eyes, dim but still attentive, followed my presence. He did not ask for tests or interventions. He asked for water. He asked for someone to hold his hand. More than anything, he asked not to be left alone.

I had been told not to touch patients. Protocols exist for a reason. Yet, in that moment, the distance those rules imposed felt greater than the risk they sought to prevent. I held his hand.

It is striking how, at the threshold of life, the complexity of medicine often dissolves into gestures that are deceptively simple. Early in training, one is taught to intervene – to diagnose, correct, stabilize. To act. Yet there are moments when none of these are central. What is required cannot be found in textbooks. It resides in the capacity to remain.

To remain without urgency. Without the illusion of control. Without framing the end as failure.

Medicine has long built its identity around the capacity to overcome disease. But there is another dimension of practice that becomes visible only when this expectation no longer holds. When cure is no longer possible, care does not disappear. It changes form.

In this sense, palliative care does not represent withdrawal. It represents a reorientation. Not toward prolonging life at any cost, but toward attending to the life that remains – its pain, its meanings, its silences.

His hand was cold, fragile, but unmistakably alive. In that hand was a life I could not fully know: relationships, work, losses, memories. It became difficult not to reflect on how often clinical practice reduces persons to beds, to numbers, to diagnoses. Efficiency can coexist with a quiet erosion of attention.

He looked at me again. He said nothing.

But the silence carried a clear request: *see me*.

And, for a moment, I did.

Not as a patient in decline, but as a person whose needs had shifted. He did not require more interventions. He required dignity. He did not ask to live longer. He asked, in a way that did not need words, to suffer less.

When his breathing slowed, the room seemed to follow. The urgency that defines hospital care gave way to a different rhythm. There was no crisis to resolve. Only a life approaching its end.

I eventually had to leave. The routines of care continued. But the question remained: what, in fact, had taken place there?

I had not altered the course of disease. I had not prevented death. Yet it did not feel like failure.

For the first time, it became clear that death is not necessarily the opposite of care. As Cassell has argued, suffering arises when the integrity of the person is threatened, not merely when disease progresses (Cassell 1982). In that room, what was at stake was not only physiology, but recognition.

To care, in that moment, was not to intervene, but to remain present in a way that preserved the person from becoming invisible.

This understanding echoes what narrative approaches to medicine have long suggested: that illness disrupts not only the body, but the story through which a person understands themselves (Charon 2006). When that story begins to fragment, the role of care may shift – from restoring coherence to bearing witness.

Looking back, what stayed was not the clinical picture, but the encounter.

There is a form of care that does not prevent death, but prevents abandonment.

And perhaps what remains, in the end, is presence.
